# Region-Specific Alterations in the Trabecular Meshwork in High- and Low-Tension Glaucoma

**DOI:** 10.7759/cureus.108581

**Published:** 2026-05-10

**Authors:** Aparna Rao, Kalindi C Muduli, Soumya Sucharita

**Affiliations:** 1 Glaucoma, L V Prasad Eye Institute (LVPEI), Mission for the Transformation of Communities (MTC) Campus, Bhubaneswar, IND; 2 Pathology, L V Prasad Eye Institute (LVPEI), Dalmia Pathology Services,, Bhubaneswar, IND; 3 Pathology, L V Prasad Eye Institute (LVPEI), Dalmia Pathology Services, Bhubaneswar, IND

**Keywords:** high-pressure glaucoma, histopathology, immunohistochemistry, low-pressure glaucoma, trabecular meshwork

## Abstract

Objective: This study evaluates histomorphometric and immunohistochemical changes in the ex vivo trabecular meshwork (TM) of high-pressure glaucoma (HPG) and normal tension glaucoma (NTG) compared to controls from donor corneoscleral buttons.

Methodology: TM specimens were collected from patients with primary open-angle glaucoma (POAG), primary angle-closure glaucoma (PACD), pseudoexfoliation glaucoma (XFG), juvenile open-angle glaucoma (JOAG), and normal-tension glaucoma (NTG) who underwent ab-interno trabeculectomy (2020-2023). Controls were obtained within three to six hours postmortem. The TM beam width in each zone was compared qualitatively with controls and between groups. At the same time, nucleated cell count and epithelial-mesenchymal transformation (EMT) changes were analyzed using ImageJ. Immunohistochemistry was used to identify expression of markers such as angiopoietin-like 1 (ANGPTL1), R-spondin 2 (RSPO2), lymphatic vessel endothelial hyaluronan receptor 1 (LYVE1), alpha-smooth muscle actin (α-SMA), and Collagen IV (COLL4) in TM regions across different glaucoma cases and controls.

Results: JOAG, POAG, and PACD exhibited reduced TM beam width, lower nucleated cell counts, and EMT changes in the CSM > JCT regions compared with controls, with JOAG showing the greatest cell loss. XFG and NTG exhibited preserved JCT nucleated cell counts with maximum ANGPTL1 expression in the JCT region in these eyes. Pronounced EMT changes and α-SMA/COLL4 or LYVE1 expression were seen in all glaucoma cases/controls, while RSPO2 expression was seen only in glaucoma.

Conclusions: Distinct histopathological and immunohistochemical changes in TM regions across HTG and NTG suggest different molecular mechanisms of TM damage.

## Introduction

The trabecular meshwork (TM) is a sieve-like structure essential for aqueous humor filtration, playing a critical role in regulating intraocular pressure (IOP) and maintaining overall ocular health [1,2]. Structural or functional damage to the TM disrupts aqueous outflow, leading to increased IOP and, ultimately, glaucoma with irreversible optic nerve damage [1-6]. The TM consists of three regions, with the juxtacanalicular meshwork being the primary site of resistance compared to the uveoscleral or corneoscleral meshwork [1,3,5-7]. TM dysfunction is a common pathological feature across various types of glaucoma, including ocular hypertension or primary open-angle glaucoma (POAG), primary angle-closure disease (PACD), pseudoexfoliation with or without glaucoma (XFG), juvenile open-angle glaucoma (JOAG), and normal-tension glaucoma (NTG) [1,3,5-6,8]. While NTG, a form of low-pressure glaucoma (LPG), is primarily associated with IOP-independent mechanisms of TM damage, IOP-induced mechanical stress remains the dominant factor in high-pressure glaucoma (HPG), including POAG, PACD, JOAG, and XFG [4-8].

The TM is anatomically and functionally divided into distinct regions, including the corneoscleral meshwork (CSM) and the juxtacanalicular tissue (JCT). Each region exhibits unique cellular and extracellular characteristics, which may be selectively impacted by specific glaucoma subtypes. Previous studies have described key ultrastructural features of glaucomatous TM, such as collapse of trabecular beams, fibrosis, extracellular material accumulation, and senile plaque deposition [5-7]. Histopathological findings include reduced intertrabecular spaces, decreased cellularity, and thinning of TM beams. However, most studies have been conducted on TM sections obtained from routine external trabeculectomies, which often include the adjacent scleral or corneal tissue, limiting their specificity for detecting changes in different regions of the tissue. Additionally, TM samples from enucleated eyes (removed for various ocular pathologies) do not accurately represent in vivo disease stages. They may be affected by post-mortem changes, leading to overlapping histopathological alterations across glaucoma subtypes. The mechanisms of TM damage in different glaucoma types remain incompletely understood, particularly the distinctions between ischemia-related damage in NTG and IOP-induced damage in HPG. The differential expression of TM markers across different regions and glaucoma types has the potential to provide valuable insights into disease mechanisms and identify biomarkers for prognosis and therapeutic targets. Isolating specific components of the outflow pathway, such as the TM, has been challenging due to its complex structure and proximity to adjacent tissues like the cornea, sclera, and uvea. Consequently, conducting robust molecular analyses of the cellular components of the TM has remained difficult until now. Recent surgical advances, such as minimally invasive glaucoma surgery (MIGS), have enabled the acquisition of pure TM tissue without adjacent structures in ex vivo samples. Additionally, advances in molecular technology, like scRNA sequencing, have identified region-specific markers within the TM, which enable delineation of each part of the outflow pathway. It's structural and functional changes in various types of glaucoma. Such techniques have identified 19 types of cells in the TM and characterized region-specific markers in TM tissue from normal cadaver donor eyes. Yet, the regional expression patterns of TM-specific markers in different types of glaucoma have not been studied earlier.

Our technique of minimally invasive ab interno trabeculectomy provides an improved method for obtaining TM samples with minimal contamination from surrounding tissues, preserving TM architecture and allowing for a more precise evaluation of structural changes across different glaucoma types [8,9]. This study aims to assess TM alterations and histopathological changes in age-matched patients with HPG forms (POAG, PACD, and XFG) and NTG, as well as in control TM obtained from donor corneoscleral tissues. We also investigated the expression patterns of key TM markers - angiopoietin-like 1 (ANGPTL1), R-spondin 2 (RSPO2), lymphatic vessel endothelial hyaluronan receptor 1 (LYVE1), and alpha-smooth muscle actin (α-SMA) - in TM specimens from patients with HPG or NTG to identify differences in molecular marker expression in TM regions between the two groups. This will enable us to understand key differences in TM damage between HPG and NTG while exploring regions that are differentially affected across glaucoma types.

## Materials and methods

All patients with POAG, PACD, XFG, NTG, or JOAG undergoing ab interno trabeculectomy with or without cataract surgery, whose TM specimens procured by ab interno trabeculectomy and sent for histopathological processing from 2020 to December 2023, were included in the study. The study was approved by the Institutional Review Board (IRB) and adhered to the tenets of the Declaration of Helsinki. Written informed consent was obtained from all patients who underwent surgery.

**Figure 1:**
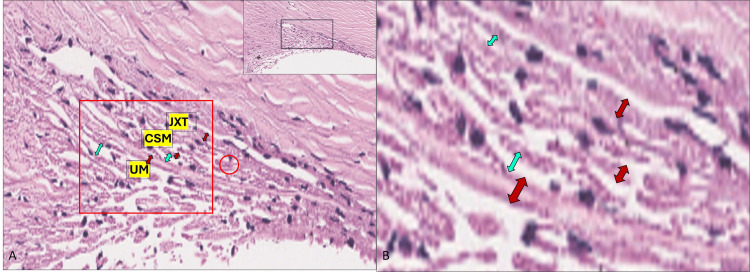
Histomorphology of the trabecular meshwork (TM). (A) A magnified image of age-matched control corneoscleral buttons from cadaveric donor eyes. TM beam width (red solid double-headed arrow) is maximal in the uveal meshwork (UM), corneoscleral meshwork (CSM), and juxtacanalicular tissue (JCT), with flatter beams in the UM. Intertrabecular (IT) spaces (blue solid double-headed arrows) are greater in the CSM than in the JCT and are reduced in areas of fusion of TM beams. (B) A higher magnification of the boxed region in (A), highlighting structural differences across the JCT, CSM, and UM regions of the TM.

The technique for TM harvesting has been described elsewhere [4,8,9]. This technique ensures retrieval of the entire TM with minimal distortion of TM architecture, allowing evaluation of alterations in different regions of the TM. Since any in vivo surgical technique may itself potentially alter the TM beams and their relationship with Schlemm’s canal (SC), we chose to evaluate histopathological specimens of corneoscleral buttons with intact TM/SC anatomy to reflect the true structure of the TM, including TM beam width and intertrabecular spaces. Therefore, for controls, instead of procuring pure TM strips from corneoscleral buttons, we used whole corneoscleral rims obtained within 3-6 hours of death from the institutional eye bank, which were sectioned to reveal TM tissue on histopathological examination (Figure 1) [6].

The cadaver buttons were cut into three pieces each (approximately 4 clock hours each), yielding 18 tissues from six cadaver donors to match the size of tissues harvested from cases (approximately 4 clock hours) and thereby avoid bias in cell counts due to the greater length of control rims compared with the segmental surgical specimens. This standardization was necessary to ensure comparability of cell counts and other variables between cases and controls (Figure 1). TM tissue harvested from cases or controls was placed in formalin vials and sent to the laboratory for histopathological analysis.

Histopathology sample processing and image analysis

The procedure for downstream processing of TM tissue obtained from surgical cases has been described in detail previously [4]. This technique ensures correct orientation to visualize all three TM regions and minimizes artifacts. Briefly, formalin-fixed tissues were paraffin-embedded with the sample oriented vertically to ensure that each TM region is represented in every section. Routine hematoxylin and eosin staining was performed, followed by examination under a light microscope (BX51, Olympus Corporation, Tokyo, Japan). Images were captured using a Magcam MU2A 2MP ½ CMOS sensor digital microscope camera mounted on an Olympus CX43 microscope (Olympus Corporation, Tokyo, Japan). Only specimens in which all three TM regions were identifiable were included for histopathological and immunohistochemical analysis. Specimens showing processing artifacts or tissue disruption - particularly in the CSM or JCT regions - were excluded.

Images obtained at 20× or 40× magnification were used for qualitative analysis of TM beam width and quantitative cell counting using ImageJ (https://imagej.nih.gov/ij/). Images were de-identified and re-numbered to ensure blinding of the observer performing the counts. Cell counting was performed using the cell counter plugin, with manual annotation of each identified cell; no calibration was required. Counts were performed three times per section per high-power field and then averaged for each tissue section to minimize error.

All three TM regions were identified in controls (Figure 1), as well as in TM specimens obtained from surgical cases, as described earlier. A qualitative assessment of TM beam morphology (beam fragmentation, thinning, and structural changes) and widening or reduction of intertrabecular (IT) spaces was performed as described previously (Figure 1) [4].

The number of nucleated cells and cells showing epithelial-mesenchymal transition (EMT) features - defined as elongated spindle-shaped nuclei with an aspect ratio > 2 - were quantified using the ImageJ cell counter function, which allows simultaneous annotation of each counted cell during manual counting [4].

Statistical analysis

While qualitative changes were analyzed descriptively, quantitative comparisons of cell counts in TM regions were performed using the unpaired Student’s t-test. Clinical and demographic variables, such as age, were compared between groups using the Kruskal-Wallis test. All analyses were performed using Stata (version 13, StataCorp, College Station, TX), with statistical significance set at *P* < 0.05.

Immunohistochemistry

We also performed immunohistochemistry on TM tissues from 32 patients (6 NTG, 6 PACD, 11 POAG, and 9 XFG) and 6 control TM specimens from cadaveric donor corneoscleral tissues. Evaluated markers included ANGPT1, specific for the juxtacanalicular tissue (JCT); RSPO2, a marker of TM beams; LYVE1, indicative of lymphatic/Schlemm’s canal endothelial cells; α-SMA, a fibroblast-specific marker; and COL1, a marker of collagen in TM beams. Samples were stratified by glaucoma type and severity to determine region- and disease-specific expression patterns.

Immunohistochemical analysis of RSPO2, ANGPT1, Collagen IV, LYVE1, and α-SMA was performed on formalin-fixed, paraffin-embedded (FFPE) tissue sections mounted on poly-L-lysine-coated slides. A polymer-based two-step indirect staining method was used in accordance with the manufacturer’s instructions.

The antibodies used included human RSPO2 (R&D Systems, Minneapolis, MN), LYVE1, Collagen IV, and ANGPT1 (Abclonal Technology, Woburn, MA), and α-SMA (Agilent Technologies, Santa Clara, CA). A secondary antibody (Quartett Immunodiagnostika & Biotechnologie, Germany) was used for signal amplification.

Tissue sections of 3-4 µm thickness were prepared and mounted on a warming table at 60-65 °C for 30 minutes. Subsequently, the sections were deparaffinized in xylene and rehydrated through a graded alcohol series. Antigen retrieval was performed using heat-induced epitope retrieval (HIER) in a Tinto Retriever system for 15 minutes at 100 °C, followed by cooling to room temperature. The staining protocol began with washing the slides in distilled water and wash buffer. Endogenous peroxidase activity was then blocked for 6 minutes, followed by an additional wash step.

Primary antibodies were applied to the tissue sections and incubated overnight at 2-8 °C, except for α-SMA, which was incubated for 1 hour at room temperature. The primary antibodies were diluted at 1:200 (1 µL antibody in 199 µL of 1× phosphate-buffered saline), except for α-SMA, which was provided as a ready-to-use formulation. Following primary antibody incubation, a primary antibody enhancer was applied for 15 minutes, followed by horseradish peroxidase for 30 minutes and 3,3′-diaminobenzidine (DAB) substrate for 8 minutes. Slides were washed with wash buffer for 5-10 minutes after each step. Finally, sections were counterstained with hematoxylin for 30 seconds, rinsed under running tap water, dehydrated through graded alcohol concentrations, and cleared in xylene. The slides were then prepared for microscopic examination.

The expression of markers in different regions and across different types of glaucoma was analyzed quantitatively using ImageJ. Thresholding in regions of interest was performed using the “Analyze Particles” function, and mean values were categorized as negative (0/-), weak (1/+), moderate (2/++), strong (3/+++), or intense/diffuse (4/++++). These assessments were performed across the TM using ImageJ. The evaluation of IHC markers focused on their expression in the JCT and CSM regions, correlating with cellularity and epithelial-mesenchymal transition (EMT) changes observed on histopathology.

## Results

The study included a total of 168 TM specimens from 166 patients, comprising 118 males and 48 females with a mean age of 46 ± 21.5 years. Of these, 51 eyes with mixed or uncertain etiologies at presentation (e.g., PACD combined with XFG), or those in which tissues showed artifacts or pigmentation that prevented clear identification of TM regions (*n* = 38), or in which tissue IDs were misplaced during processing (*n* = 4), were excluded from the study. Ultimately, 75 TM specimens were analyzed, including 30 POAG, 17 PACD, 15 XFG, 9 NTG, and 4 JOAG cases (Table 1). All TM samples were harvested and immediately fixed in formalin for histopathological analysis using hematoxylin and eosin (H&E) staining.

**Table 1:** Clinical and demographic details of patients with high-pressure or low-pressure glaucoma undergoing microincisional trabeculectomy. POAG, primary open-angle glaucoma; PACD, primary angle-closure disease; JOAG, juvenile open-angle glaucoma; XFG, exfoliation glaucoma; NTG, normal-tension glaucoma; TM, trabecular meshwork; SD, standard deviation; #, control (TM from donor corneoscleral buttons from the eye bank; see text for full description); *, significance shown only when *P* < 0.05 compared to JOAG, with no difference between POAG, PACD, XFG, NTG, or controls; ^, Kruskal-Wallis test.

	POAG (Mean ± SD) (N = 30)	PACD (Mean ± SD) (N = 17)	JOAG (Mean ± SD) (N = 4)	XFG (Mean ± SD) (N = 15)	NTG (Mean ± SD) (N = 9)	Controls^#^ (Mean ± SD) (N = 6)	P-value^
Age (years)	48 ± 10.7	51 ± 9.8	32 ± 12.2	50 ± 12.6	49 ± 9.5	58 ± 10.2	0.03*
IOP at surgery (mmHg)	28 ± 5.2	22 ± 10.4	32 ± 6.7	27 ± 10.6	18 ± 3.6	NA	<0.001
Visual field mean deviation at surgery (dB)	-16 ± 6.8	-14 ± 4.9	-19 ± 9.2	-17 ± 8.9	-14 ± 5.4	NA	NS

**Figure 2:**
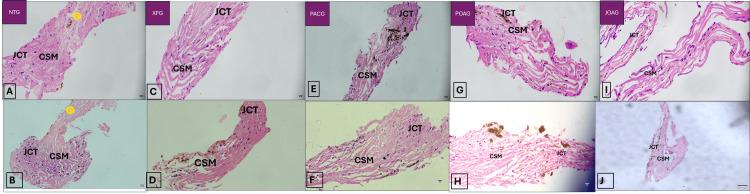
Light microscopy of the TM from NTG showing expanded uveal zone (orange circle; A, B) with visual field mean deviation of -8.5 dB and -10.6 dB, respectively; XFG (C, D; MD -9.2 and -10.3 dB, respectively); PACD (E, F; MD -7.1 and -14.6 dB, respectively); POAG (G, H; MD -13.7 and -12.3 dB, respectively); and JOAG (I, J; MD -14.7 and -15.8 dB, respectively). Changes in glaucoma cases included reduced TM beam width in both the JCT and CSM regions, reduced cell counts, and EMT changes. NTG, normal-tension glaucoma; XFG, exfoliation glaucoma; PACD, primary angle-closure glaucoma; POAG, primary open-angle glaucoma; JOAG, juvenile open-angle glaucoma; TM, trabecular meshwork; CSM, corneoscleral meshwork; JCT, juxtacanalicular tissue; EMT, epithelial-mesenchymal transition

The CSM occupied the majority of the TM, comprising approximately 50%-70% of the TM tissue, with JCT width being maximal in NTG eyes (Table 2, Figures 1-2).

**Table 2:** Qualitative changes on histopathological sections of trabecular meshwork obtained from patients with high- or low-tension glaucoma. ^#^Controls mean specimens from eye bank corneoscleral buttons. *The UM zone was not identified in three JOAG specimens. POAG, primary open-angle glaucoma; PACD, primary angle-closure glaucoma; JOAG, juvenile open-angle glaucoma; XFG, exfoliation glaucoma; NTG, normal-tension glaucoma; TM, trabecular meshwork; CSM, corneoscleral meshwork; JCT, juxtacanalicular tissue; UM, uveal meshwork

Type of glaucoma	TM beam width in the CSM region (µ)	TM beam width in the JCT region (µ)	Width of the UM zone in TM tissue (µ)
PACD	Reduced	Reduced	Reduced
POAG	Reduced	Reduced	Reduced
JOAG	Reduced	Reduced	Reduced
XFG	Reduced	Reduced least	Reduced
NTG	Comparable to controls or minimally reduced	Comparable to controls or minimally reduced	Increased

The intertrabecular spaces were narrower in the JCT region compared to the CSM zone in controls (Figures 1-2).

The uveal meshwork was not discernible in 56 TM tissues obtained from cases, since the surgical technique involves dissecting the adhesions of the iris from the TM, which may sever the UM in some cases. We now analyzed TM morphology across the three regions and compared it with controls.

Qualitative differences in TM morphology between cases and controls

All glaucoma types exhibited thinning of TM beams and altered architecture of the CSM and JCT regions compared with controls (Figure 2). JOAG, PACD, and POAG eyes exhibited the most pronounced thinning of TM beams compared with controls, in both the CSM and JCT regions. The thinned TM beams appeared rounded and often resembled single fibers rather than the typical broad structure observed in controls (Figure 2). In some areas, TM beams in the CSM region of POAG or XFG eyes appeared to fuse, resulting in a spurious increase in beam thickness and loss of cellular nuclei (Figures 1-2).

Morphometric differences in NTG vs. high-tension glaucoma forms (POAG, PACD, XFG, JOAG)

NTG

TM beam width was least affected in the CSM and JCT regions compared with HPG forms and was comparable to controls (Table 2, Figure 2). The UM width was widest in NTG eyes compared with both controls and high-tension glaucoma (HTG) (Figure 2). Cell counts were higher in NTG compared with HPG in the CSM (36 ± 23.4 µm) and JCT (32 ± 16 µm) regions, compared with 19 ± 17.1 µm in NTG and 17 ± 6.4 µm in PACD, respectively (Table 3). In contrast, EMT changes were more pronounced in the CSM region despite only a minimal reduction in cell counts compared with other HPG forms (Figure 2). This suggests that TM cell counts are relatively preserved in NTG compared with HPG, although TM dysfunction and EMT changes are observed in both HPG and NTG eyes (Tables 2-3).

**Table 3:** Cell count and functional changes in histopathology in the trabecular meshwork obtained from high-pressure or low-pressure glaucoma patients after microincisional trabeculectomy. ^#^EMT changes refer to the number of cells and not the extent or severity of EMT changes. *P < 0.05, *P*-values indicated as above. POAG, primary open-angle glaucoma; PACD, primary angle-closure glaucoma; JOAG, juvenile open-angle glaucoma; XFG, exfoliation glaucoma; NTG, normal-tension glaucoma; TM, trabecular meshwork; CSM, corneoscleral meshwork; JCT, juxtacanalicular tissue; EMT, epithelial-mesenchymal transformation; NA, not applicable since these cells do not have comparative variables

	Cell count in the CSM zone	Cell count in the JCT zone	*P*-value (t-test for difference between CSM and JCT)	EMT changes^#^ in the CSM zone	EMT changes in the JCT zone	*P*-value (t-test for difference between CSM and JCT)
Controls	45±12.4	55 ± 12.9	<0.0001^*^	8 ± 2.3	3 ± 1.2	<0.0001
PACD	19 ± 17.1	17 ± 6.4	<0.0001^*^	14 ± 13.7	7 ± 7.9	<0.0001
POAG	26 ± 31.3	24 ± 12.05	<0.0001^*^	16 ± 12.09	5 ± 6.08	<0.0001
JOAG	26 ± 16.2	12 ± 1.4	<0.0001^*^	20 ± 15.5	6 ± 4.2	<0.0001
XFG	22 ± 5.03	52 ± 16.6	<0.0001^*^	13 ± 2.1	2 ± 2.1	<0.0001
NTG	36 ± 23.4	32 ± 16.5	<0.0001^*^	28 ± 22.3	12 ± 6.5	<0.0001
*P*-value (Kruskal-Wallis for between groups)	<0.0001^*^	<0.0001^*^	NA	0.03^*^	<0.0001^*^	NA

All HPG forms, compared with NTG, exhibited thinning of TM beams in both the CSM and JCT regions compared with controls (Table 2). In XFG, reductions in cell counts and EMT changes were predominantly seen in the CSM region, with minimal changes in both parameters in the JCT region compared with controls or other HPG forms (Tables 2-4). JOAG eyes showed the maximal reduction in cell counts among all HPG forms (Table 2, Figure 2), markedly thin TM beams, and a higher proportion of EMT changes in both the CSM and JCT regions. The cell count in the JCT region was 12 ± 1.4 compared with 24 ± 12.05 in POAG and 55 ± 12.9 in controls (*P* < 0.001; Table 2). These findings suggest a more global and severe response to intraocular pressure (IOP) in JOAG.

Regional expression of immunohistochemical markers in the TM in relation to morphological changes

**Figure 3:**
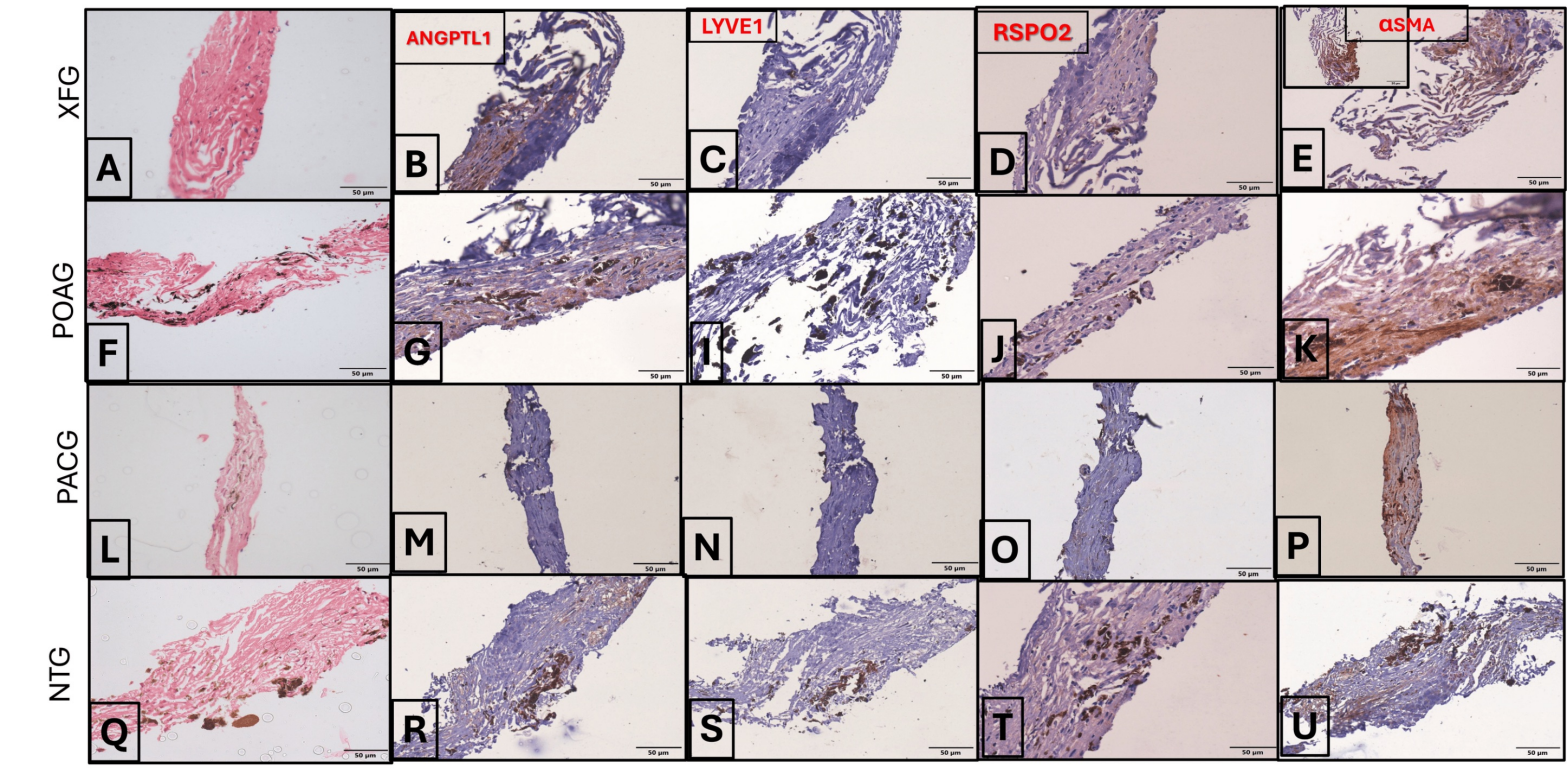
Immunohistochemical expression of different markers across TM regions in XFG (A-E), POAG (F-K), PACD (L-P), and NTG (Q-U). ANGPTL1 expression was maximal in the JCT region in cases. LYVE1 expression was seen in regions of EMT in the CSM and JCT regions in all cases, with maximal changes in XFG and least expression in PACD eyes. RSPO2 expression was observed in both CSM and JCT regions, with minimal expression in PACD eyes. α-SMA expression was diffusely present across all regions in all cases. ANGPTL1, angiopoietin-like 1; LYVE1, lymphatic vessel endothelial hyaluronan receptor 1; RSPO2, R-spondin 2; α-SMA, alpha-smooth muscle actin; TM, trabecular meshwork; XFG, exfoliation glaucoma; POAG, primary open-angle glaucoma; PACD, primary angle-closure disease; NTG, normal-tension glaucoma; CSM, corneoscleral meshwork; JCT, juxtacanalicular tissue; EMT, epithelial-mesenchymal transition

Immunohistochemistry was performed on TM tissues from 32 of 168 patients (6 NTG, 6 PACD, 11 POAG, and 9 XFG) and 6 control TM specimens from cadaveric donor corneoscleral tissues. Immunohistochemical marker expression also varied across different TM regions and glaucoma types (Figure 3).

ANGPTL1 staining was pronounced (++++) and predominantly restricted to the JCT region in controls and all glaucoma types (Figure 3), with maximal expression in XFG. Its expression was least in PACD eyes (Table 3).

α-SMA expression, seen in the extracellular region around the TM beams, was consistently diffuse across the entire TM. Strong expression was seen in all primary glaucoma types, including NTG, POAG/OHT, PACD, and XFG (Tables 3-4). Collagen IV showed a diffuse expression pattern (++) across all subtypes on the surface of TM beams in the JCT and CSM regions (Figure 3).

**Table 4:** Regional expression of specific markers in the TM in patients with high-pressure glaucoma or NTG. POAG, primary open-angle glaucoma; PACD, primary angle-closure glaucoma; JOAG, juvenile open-angle glaucoma; XFG, exfoliation glaucoma; NTG, normal-tension glaucoma; TM, trabecular meshwork; CSM, corneoscleral meshwork; JCT, juxtacanalicular tissue; EMT, epithelial-mesenchymal transformation; ANGPTL1, angiopoietin-like 1, RSPO2, R-spondin 2; LYVE1, lymphatic vessel endothelial hyaluronan receptor 1; α-SMA, alpha-smooth muscle actin

Region	Glaucoma type	ANGPTL1	RSPO2	LYVE1	COLL4/α-SMA
JCT	Controls	++++ (Diffuse)	- (Absent/mild)	-/+ (Absent/mild)	++++ (Diffuse)
	XFG	++++ (Diffuse)	++ (Moderate)	+++ (Diffuse)	++++ (Diffuse)
	POAG	++ (Moderate)	++ (Moderate)	++ (Moderate)	++++ (Diffuse)
	NTG	++ (Moderate)	++ (Moderate)	+++ (Diffuse)	++++ (Diffuse)
	PACD	+++ (Moderate)	+/++ (Mild-moderate)	+ (Mild)	++++ (Diffuse)
CSM	Controls	- (Absent)	++ (Moderate)	-/- (Absent/mild)	++++ (Diffuse)
	XFG	- (Absent)	++ (Moderate)	+++ (Diffuse)	++++ (Diffuse)
	POAG	- (Absent)	++ (Moderate)	++ (Moderate)	++++ (Diffuse)
	NTG	- (Absent)	++ (Moderate)	+++ (Diffuse)	++++ (Diffuse)
	PACD	- (Absent)	+ (Mild)	+ (Mild)	++++ (Diffuse)

RSPO2 expression was predominantly localized and minimal in controls, and was observed in all cases in the CSM region (Figure 3).

LYVE1 expression was prominent in regions with severe EMT changes and was seen in both the CSM and JCT regions across all glaucoma types, with maximal expression in XFG and NTG, and least expression in PACD eyes (Figures 2-3).

Table 4 summarizes the changes across HPG forms, controls, and NTG eyes.

## Discussion

This study identified significant structural changes in the TM, particularly TM thinning and reduced TM cell counts, predominantly in JOAG and PACD among high-pressure glaucoma forms. To summarize, IOP-induced changes were predominantly seen in the CSM regions > JCT region in the form of reduced TM beam width in HPG forms. This was associated with reduced cell counts in POAG, PACD, and JOAG. EMT changes were more predominant in the JCT region in NTG and HPG forms, with minimal reduction in cell count in the JCT region in NTG and XFG, comparable to controls. JOAG eyes had a global response, with all regions severely affected compared to other HPG forms. These findings suggest that the type of TM dysfunction induced by ischemia in NTG and IOP-induced changes in HPG forms are distinct. These patterns point to variability in regional remodeling in TM tissue across HPG and NTG.

Among the HPG types, XFG, known to have both IOP-induced changes and ischemic risk factors, exhibited relative sparing of JCT cellularity compared to other HPG forms, which was confirmed on IHC. The JCT may be more sensitive to ischemic triggers, being close to the endothelium lining Schlemm’s canal. Previous studies have extensively analyzed TM ultrastructure and histopathological features in various glaucoma types and aging eyes [4,6,9,10-20]. However, to the best of our knowledge, no prior research has systematically examined region-specific TM changes in HPG and NTG [10,16,21-23]. Tamm et al. have extensively characterized ECM accumulation in the JCT as a key contributor to outflow resistance in POAG; however, our findings indicate relative JCT cell count sparing in XFG and NTG, suggesting alternative mechanisms of TM dysfunction beyond traditional ECM alterations [4,16,18,23]. Transforming growth factor beta (TGF-β) mediated fibrosis is known to mediate the cascade of events typical of glaucoma [23]. TM beam thinning predominantly in the CSM regions in HPG indicates increased extracellular matrix deposition, potentially leading to outflow resistance. These findings suggest that TM cells in the CSM region may have a sensor role in detecting IOP-induced mechanical stretch, potentially mediating IOP-related changes in POAG, PACD, JOAG, and XFG. Differential signatures in cells of the JCT and CSM regions in each glaucoma form may provide insights into distinct molecular processes triggered by raised IOP versus those triggered by ischemia.

The role of LYVE1 appears particularly relevant here, as its expression was prominent in regions with severe EMT changes, including the CSM in XFG. LYVE1-positive macrophages are present in the eye, and their role in the eye and glaucoma has been reported in several studies, highlighting their function in tissue homeostasis and repair. Though first identified as a marker of lymphatic endothelial cells, they are now linked to a subset of resident macrophages that regulate tissue fibrosis and repair. Its role in maintaining TM function and ECM remodeling may be crucial to understanding the phenotypic differences between POAG, PACD, and XFG, where exfoliative material deposition in the TM regions activates macrophages and upregulates ECM degradation processes. Additionally, LYVE1, a key marker of lymphatic endothelial cells, is expressed in Schlemm’s canal, and its increased presence in NTG and XFG may reflect a compensatory mechanism for lymphatic-like drainage of accumulated debris or a response to ischemia. Oxidative stress, which plays a major role in NTG and XFG pathology, may also promote LYVE1 expression in these eyes. Furthermore, since LYVE1 binds hyaluronan, a crucial ECM component, its upregulation in XFG could be linked to alterations in hyaluronan metabolism and an attempted homeostatic response to chronic ischemic or oxidative stress in NTG. These processes, therefore, differ from the IOP-induced changes in POAG that may determine the clinical behavior of each glaucoma type.

The role of ANGPTL1 in glaucoma and JCT function is now increasingly being recognized. This marker was prominently expressed in the JCT region in most HPG forms and NTG eyes. Its role in TM function is not clearly understood. ANGPTL1 knockout mice exhibit reduced basal IOP by approximately 2 mmHg compared with wild-type mice, with a similar trend observed in heterozygotes. In contrast, elevating ANGPTL1 levels in murine eyes through injections leads to an increase in IOP. Excess ANGPTL1 is reported to increase outflow resistance and IOP, with beneficial effects seen with neutralization of this marker in both naïve and steroid-induced hypertensive eyes. High expression in the JCT region in XFG suggests differential responses of the JCT region, which may respond more to ischemic triggers, while the TM beams in the CSM region may mediate IOP-induced changes in TM function. These differential mechanisms may hold the key to different clinical phenotypes and behaviors in different glaucoma forms.

This study had several limitations. While our study provides insights into regional TM morphological and immunohistochemical changes across glaucoma subtypes, we did not perform severity-matched comparisons due to the relatively small sample size in some types, such as JOAG. Although measurements were performed repeatedly, some degree of observer bias cannot be excluded; however, we believe this was offset by the use of automated cell counting with ImageJ and blinding of clinical details. As our primary objective was to compare expression between HPG and NTG, we included both forms of glaucoma (POAG, PACD, and XFG vs. NTG), along with JOAG, to assess differences in expression not only between glaucoma subtypes but also in relation to potential age-related effects. We did not perform quantitative analysis of sections; qualitative changes can differ postmortem, and although fixation of tissue in formalin immediately after harvest minimizes artifacts, these cannot be completely avoided, precluding reliable quantitative analysis. We could not perform immunohistochemistry in JOAG samples owing to severe changes and reduced cellularity. Further, these are preliminary results, which will require future studies in each glaucoma group with additional markers, quantitative analysis, transcriptomics, and functional tests to identify the role of each marker in TM damage in glaucoma.

## Conclusions

Nevertheless, our study demonstrates distinct structural histological changes and region-wise expression of different TM markers in different types of glaucoma. Region-specific TM susceptibilities and tissue responses to IOP versus other triggers may possibly explain HPG and NTG clinical phenotypes. Future studies investigating TM cell molecular signatures and functional studies on ANGPTL1 and LYVE1 in TM dysfunction across different glaucoma types may provide deeper insights into primary glaucoma pathogenesis and potential therapeutic targets.
